# Functional and morphological cardiovascular alterations associated with neurofibromatosis 1

**DOI:** 10.1038/s41598-020-68908-0

**Published:** 2020-07-21

**Authors:** Antonio Cutruzzolà, Concetta Irace, Marco Frazzetto, Jolanda Sabatino, Rosa Gullace, Salvatore De Rosa, Carmen Spaccarotella, Daniela Concolino, Ciro Indolfi, Agostino Gnasso

**Affiliations:** 1 Dipartimento di Medicina Sperimentale e Clinica, University Magna Græcia, Viale Europa Località Germaneto, 88100 Catanzaro, Italy; 2 Dipartimento di Scienze della Salute, University Magna Græcia, Catanzaro, Italy; 3 Dipartimento di Scienze Mediche e Chirurgiche, University Magna Græcia, Catanzaro, Italy; 4 Center of Cardiovascular Research, University Magna Graecia, Mediterranea Cardio Centro, Catanzaro, Napoli, Italy

**Keywords:** Cardiology, Risk factors

## Abstract

Subjects with Neurofibromatosis 1 (NF1) develop vascular complications. The protein product of the gene affected in NF1, neurofibromin, physiologically modulates endothelial function and preserves vascular and myocardial structure. Our study aimed to verify whether subjects with NF1 have early, preclinical abnormalities of carotid artery structure, brachial artery function, and cardiac function. We recruited 22 NF1 subjects without previous cardiovascular events and 22 healthy control subjects. All subjects underwent measurement of carotid artery intima-media thickness (IMT), evaluation of brachial artery endothelial function after ischemia and exercise, and cardiac function. Mean IMT was 543 ± 115 μ in NF1 subjects and 487 ± 70 μ in Controls (*p* < 0.01). Endothelial function was significantly dumped in NF1 subjects. The dilation after ischemia and exercise was respectively 7.5(± 4.8)% and 6.7(± 3.0)% in NF1 versus 10.5(± 1.2)% and 10.5(± 2.1)% in control subjects (*p* < 0.02; *p* < 0.002). Left ventricular systolic function assessed by Global Longitudinal Strain was significantly different between NF1 subjects and Controls: − 19.3(± 1.7)% versus − 21.5(± 2.7)% (*p* < 0.008). These findings demonstrate that NF1 patients have early morphological and functional abnormalities of peripheral arteries and systolic cardiac impairment and suggest the need for a tight cardiovascular risk evaluation and primary prevention in subjects with NF1.

## Introduction

Neurofibromatosis 1 (NF1) is a common genetic disease, affecting approximately 1 in 3,500 subjects. It has an autosomal dominant inheritance with complete penetrance, variable expression, and a high rate of new mutations. The disease is characterized by cafe-au-lait spots, dermal neurofibromas, skeletal dysplasia, Lish nodules, and optic glioma^1^. Neurofibromin (encoded by the NF1 gene) is a modulator of cell growth, downregulates ras signaling, and its loss of function promotes cellular proliferation^2^. For these reasons, subjects with NF1 have increased risk for malignancies like peripheral nerve sheath tumor, leukemia, and rhabdomyosarcoma.

Furthermore, they may develop vascular complications as renal artery stenosis with secondary hypertension, aneurysm, arterial stenosis/occlusion, myocardial infarction, and cerebral or visceral infarcts arteriovenous fistulae^3^. The vascular involvement does not seem directly linked to the proliferation and malignant transformation of neural-crest derivatives^2^. Though pathogenesis of vascular lesions in NF1 remains unclear, previous studies in murine models and humans demonstrated that neurofibromin is expressed in the endothelial cells and vascular smooth muscle cells as well^2,4^. Therefore NF1 vasculopathy might be the consequence of a direct involvement in vascular layers^5^.

Cardiac alterations have also been under the spotlight in NF1. The neurofibromin protein is physiologically involved in cardiac development^6^. Besides, microdeletions of the NF1 gene often involve the nearby SUZ12 and CENTA2 genes, whose haploinsufficiency may also influence cardiac development^7^.

The present study aims to verify whether patients with NF1 have early abnormalities of carotid artery structure, of brachial artery, and cardiac function.

## Materials and methods

### Patients and study design

Subjects with NF1 from the regional reference center for genetic diseases at the University Magna Græcia of Catanzaro were consecutively enrolled if they met the following inclusion and exclusion criteria. Inclusion criteria: NF1 diagnosis according to the presence of two or more of the following features: cafe-au-lait spots, neurofibromas, freckling in the axillary or inguinal region, skeletal dysplasia, Lish nodules, optic glioma and a first-degree relative with NF1^8^; exclusion criteria: pregnancy, other genetic or metabolic diseases, known history of hypertension, hyperlipidemia, and diabetes, previous cardiovascular events as myocardial infarction, stroke, peripheral artery disease, angina, prior revascularization, and use of vasoactive drugs or lipid-lowering drugs, antihypertensive and antidiabetic treatment. As a control group, healthy students attending the Medical School at the University Magna Græcia Catanzaro and employees of the same Institution, age-matched, without cardiovascular risk factors or history of cardiovascular disease, were enrolled. The research was approved by the Institutional Ethics Committee 'Regione Calabria Area Centro' Protocol Number 250/December 2016, and conducted following the Declaration of Helsinki. The participants were recruited after the protocol was clearly explained, and after they signed the Informed Consent. In the case of subjects younger than 18 years old, the Informed Consent was signed by at least one parent. The study has been registered as an observational study. The visit included clinical examination, vascular studies, transthoracic echocardiography, and fasting blood sample withdrawal to measure viscosity, blood lipids, and glycemia. Systolic and diastolic blood pressure (SBP, DBP) and heart rate (HR) were measured. Body weight and height were used to calculate body mass index (BMI) as kg/m^2^. Waist was measured in the midway between the top of the hip bone and the bottom of the ribs.

### Blood withdrawal

Blood sample was withdrawn after at least 8 h of fasting. Blood lipids and fasting plasma glucose were measured. Lipids were measured with a commercially available kit and fasting plasma glucose with the glucose-hexokinase method (Roche, Basel, Switzerland).

### Blood viscosity measurement

Blood and plasma viscosity were measured at 37 °C within two h from blood withdrawal, and after adding heparin (35 U ml), by a cone-plate viscometer (Well-Brookfield DVIII, Middleboro, MA, US) equipped with a cp-40 spindle. Blood viscosity was measured at shear rate 225/s, that is the shear developing in medium and large-sized arteries^9^. In our laboratory, the coefficient of variation for blood viscosity was 2% at shear rate 225/s^10^.

### Carotid IMT

The study was performed using an echo-Doppler Philips HD 11XE (Royal Philips Electronics, the Netherlands) equipped with a 12–3 MHz linear array, steerable pulsed wave Doppler, and simultaneous ECG recording. All subjects were preliminarily examined to evaluate the presence of plaques or stenosis. A complete carotid ultrasound examination was performed to evaluate both sides internal, external, and common carotid arteries. Plaque was defined as a focal lesion encroaching into the lumen with a thickness ≥ 1.5 mm^11^. Stenoses were assessed using the grayscale and pulsed Doppler parameters, including Internal Carotid Artery (ICA) Systolic Peak Velocity (SPV), ICA End Diastolic Velocity (EDV), Common Carotid Artery (CCA) SPV, CCA EDV, peak systolic ICA/CCA ratio (SVR), and peak end-diastolic ICA/CCA ratio (EDR)^12^. The study was performed before blood withdrawal in a temperature-controlled (20–24 °C) room and after the patient was at rest for 10 min. After a preliminary scan, the probe position was adjusted, obtaining an angle between the ultrasound beam and the longitudinal axis of the vessel at 90°. The CCA was visualized in the longitudinal section, 1 cm proximal to carotid bulb. Images from three different projections (anterior, lateral, posterior) were recorded at the R-wave of the cardiac cycle for offline Intima-Media Thickness (IMT) measurement.

Mean IMT was calculated as the mean of the three projections measurement; Maximal IMT was the highest IMT value among the three projections. IMT, defined as the distance between the leading edge of the lumen-intima interface and the inner edge of the media-adventitia interface of the CCA far wall, was measured offline using dedicated software (Autodesk Design Review, BSA Italy, https://knowledge.autodesk.com/it)^13^. Common carotid artery internal diameter (ID) was also measured, at the R wave of the cardiac cycle, using the caliper of the instrument in the middle portion of the examined common carotid artery and in the three different projections. ID was defined as the distance between the intima-lumen interface of the near wall and the lumen-intima interface of the far wall. The intra-operator IMT reproducibility was evaluated ten subjects were studied twice apart, and the coefficient of correlation between two measurements was 0.98.

### Endothelial function

Endothelial function was evaluated by the Flow-Mediated Dilation (FMD) technique using two different stimuli, ischemia, and handgrip exercise (HG-EX).

Tests were performed using the same echo-Doppler previously described, and simultaneous ECG recording. Participants were asked to abstain from exercise, alcohol, caffeine, food, and smoking for 12 h before the study. Premenopausal women were assessed in the first week of the menstrual cycle to minimize the effect of sex hormones on vasodilation. FMD was evaluated in the brachial artery of the non-dominant arm, with ischemia stimulus first. The two tests were performed at least 30 min apart, and always after baseline conditions (brachial artery diameter and blood flow velocity) were restored. In both experiments, the brachial artery was imaged ~ 3–4 cm above the elbow in the longitudinal section on the anterior side of the biceps muscle keeping the angle between the ultrasound beam and the vessel at 90°.

Ischemic test was carried out by inflating a pneumatic cuff around the forearm up to 250 mmHg and maintaining inflation for 5 min. Images of the brachial artery ID at baseline, 1, 2 min, and 3 min after ischemia were recorded and analyzed offline using the software Autodesk1 Design Review^14,15^. Again, ID was defined as the distance between the intima-lumen interface of the near wall and the lumen-intima interface of the far wall. FMD was expressed as percentage change from baseline and calculated using the following formula: [(after ischemia-ID) − (baseline-ID)/(baseline-ID)] × 100. The highest FMD among those calculated at 1, 2, and 3 min was defined as peak FMD. Furthermore, based on the time of maximal dilation, subjects were divided into Early dilators (dilation at 1 min), Late dilators (dilation after 1 min), No dilators (absence of dilation). The three set-times (1, 2, and 3 min) were determined by preliminary vascular studies performed in 15 healthy subjects (no history of cardiovascular disease) aged 20–50 years. Brachial artery diameter was measured every 5 s up to 180 s after the cuff release. Seven subjects showed peak FMD between 50 and 65 s; 5 between 115 and 125 s; 3 did not show any dilation. The mean difference of maximal brachial artery diameter detected in the range from 50 to 65 s was 0.09 mm, and 0.13 mm in the range from 115 to 125 s. According to these observations, we decided to set observation times at 1, 2, and 3 min.

HG-EX test was performed with participants lying in the supine position with the left arm extended out at an angle of 80 degrees and with the handgrip dynamometer in their left hand. All subjects were instructed on how to perform a maximal voluntary contraction (MVC) and a short bout of handgrip exercise in a 2 s contraction:3 s relaxation ratio^16^. The test consisted of 6 min rhythmic isometric handgrip exercise at 30% intensity of MVC. MVC was evaluated at the beginning of the visit to avoid any interference with vascular tests. The 6 min exercise at 30% MVC was preliminarily established in healthy volunteers and represented the maximal contraction, ensuring an adequate vasodilation without systemic hemodynamic changes (heart rate and blood pressure) and pain or fatigue. Brachial artery ID was measured at rest before exercise and every min from 1st to 6th min. HG-EX FMD was expressed as percentage change from baseline and calculated using the following formula: [(1–2–3–4–5–6-min HG-EX ID) − (baseline-ID)/(baseline-ID)] × 100.

Both stimuli explore brachial artery endothelium-dependent dilation by increasing peripheral metabolic demand. Indeed, ischemia or exercise determines the decrease of peripheral resistances in the downstream vasculature such that upon the stimulus is removed, the increase in brachial artery shear stress occurs. Shear stress is the mechanical force acting on the endothelial surface and stimulating the release of vasodilator substances. Ischemic test induces a transient and vigorous increase in shear stress, while exercise a sustained increase. Exercise is a more physiological stimulus, representing what happens in everyday life^16^.

Brachial artery blood flow velocities (SPV, EDV, Mean Velocity (MV)) were automatically detected by pulsed-wave Doppler, and all scans were performed at an insonation angle of 68°, which still provides valid estimates of flow velocity as it allows the vessel to be perpendicular to the ultrasound beam, which yields superior image quality^14,17^. Velocities were recorded before and after the HG-EX.

Brachial artery blood velocities and blood viscosity were used to calculate wall shear stress (WSS). WSS was calculated at baseline and after the last bout of handgrip exercise (end exercise) throughout the cardiac cycle as peak SS (PSS), and mean SS (MSS) using the following formulas:

$$\begin{aligned} {\text{PSS dynes/cm}}^{2} & = 4\upeta \,{\text{SPV/ID}} \\ {\text{MSS dynes/cm}}^{2} & = 4\upeta \,{\text{MV/ID}} \\ \end{aligned}$$

where η is blood viscosity measured in poise, SPV in cm/s, and ID in cm^10^.

### Assessment of cardiac function

Transthoracic echocardiographic studies were performed using Vivid E95 (GE Healthcare), which was equipped with adult and pediatric transducers. All data were transferred to a commercially available workstation (EchoPAC, GE Healthcare) and analyzed offline. Chamber size and function were assessed according to the latest guidelines. Values were adjusted for body surface area. Left ventricular Ejection Fraction (EF) was calculated using the traditional Simpson method from apical 4 chamber and apical 2 chamber views. According to the practice guidelines, we used 52–72% as LVEF normal reference range for males and 54–74% for females^18^. Fractional Shortening (FS) was calculated as the fraction of Left Ventricular (LV) diastolic dimension that is lost in systole from M-Mode acquisition of parasternal long-axis views. Global Longitudinal Strain (GLS) was measured using image loops of the apical 4 chamber and apical 2 chamber views. 2-D speckle-tracking echocardiographic analyses were performed by a different experienced cardiologist (blinded to clinical data and previous examinations), using Echopac. For speckle tracking analysis, we acquired apical four-, three- and two-chamber view images using conventional two-dimensional greyscale echocardiography, during breath-hold and with a stable ECG recording. To guarantee optimal tracking, three consecutive heart cycles were obtained at a frame rate of 50–80 frames/second, sinus rhythm, and ≤ 10% variability in heart rate, as previously described^19^. We adopted the GLS normality interval used in the previous EACVI-led NORRE study, ranging from − 16.7 to − 26.7% for males and − 17.8 to − 28.2 for females^20^.

### Statistical analyses

Data were analyzed using SPSS 23 for Microsoft (SPSS, Inc., Chicago, IL). Shapiro–Wilk test was used to assess normality distribution. Variables not normally distributed were CCA ID, baseline MSS, and maximal voluntary contraction. Right and left CCA IMT were grouped for the analysis. The *t*-test for independent samples was used for data normally distributed, while for data not normally distributed Mann–Whitney U test was preferred. Pearson's Chi-square or Fisher exact test was used to compare categorical data as appropriate. Paired-samples T-test was used to evaluate differences between brachial artery diameter and WSS measured at baseline and after HG-EX in both groups. A mixed-design ANOVA (repeated measures ANOVA with a between-subject factor) was used to compare FMD measured at each minute of the HG-EX test between groups. The ANCOVA was used to evaluate the effect of possible confounders (sex, gender, and smoke) when comparing FMD between NF1 and control subjects.

## Results

Twenty-two subjects with NF1 and 22 healthy control subjects were recruited. Clinical, biochemical, and anthropometric data of the study population and clinical features associated with NF1 are shown in Table 1. No statistically significant difference was found between groups except for heart rate that was significantly higher in NF1 subjects.

**Table 1:** Characteristics of subjects included in the study.

Variable	NF1 (n 22)	Control subjects (n 22)	*p*
Age (years), 95% [CI]	29 ± 12, [12–55]	28 ± 11, [16–55]	0.64
**Age group**			
10–19	6 (27.3%)	4 (18.2%)	
20–29	6 (27.3%)	12 (54.5%)	
30–39	5 (22.7%)	3 (13.6%)	
40–49	3 (13.6%)	1 (4.5%)	
50–59	2 (9.1%)	2 (9.1%)	
Male	10(45)	14(64)	0.26
Waist (cm)	83 ± 15	82 ± 9	0.76
BMI (kg/m^2^)	24 ± 4	23 ± 3	0.13
Systolic BP (mmHg)	115 ± 8	114 ± 8	0.53
Diastolic BP (mmHg)	74 ± 8	70 ± 8	0.06
Plasma glucose (mg/dl)	84 ± 8	81 ± 9	0.25
Total cholesterol (mg/dl)	158 ± 23	156 ± 19	0.75
HDL-cholesterol (mg/dl)	57 ± 8	55 ± 8	0.41
Triglycerides (mg/dl)	57 ± 19	61 ± 24	0.54
HR (beats/min)	73 ± 10	67 ± 8	0.04
Smokers	2(10)	1(5)	0.10
Café au lait macules (%)	22 (100)	–	–
Cutaneous/subcutaneous or plexiform neurofibroma (%)	10 (45)	–	–
Lish nodules (%)	12 (55)	–	–
Bony dysplasia	7 (32)	–	–
First degree relative with NF1	4 (18)	–	–

Data are displayed as mean ± SD or n (%).

*NF1* neurofibromatosis 1, *SBP* systolic blood pressure, *DBP* diastolic blood pressure, *HR* heart rate.

**Figure 1:**
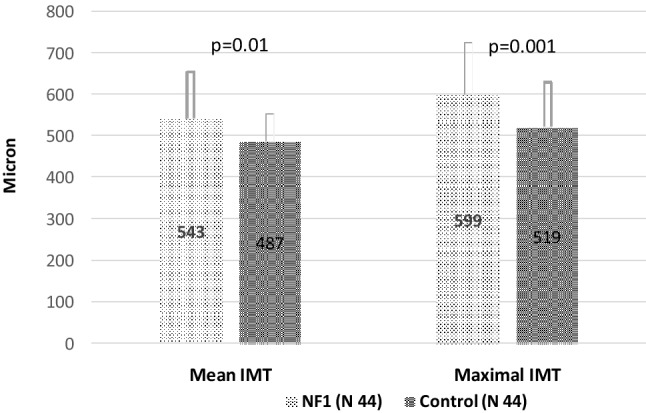
Mean and Maximal Intima-media thickness (IMT) of Common Carotid artery in NF1 subjects and control subjects.

The preliminary scan of carotid arteries excluded plaques and stenosis in the examined vascular segments (internal, external, and common carotid artery). Figure 1 shows mean and maximal carotid IMT of grouped right and left CCA in NF1 (total carotid arteries 44 per group) and Control subjects. The difference in mean and maximal IMT between groups was statistically significant (mean IMT *p* < 0.01, and maximal IMT *p* < 0.001). ID of the common carotid artery was 5.8 ± 1.3 mm in NF1 subjects and 5.9 ± 0.8 in control subjects (*p* = 0.78).

**Figure 2:**
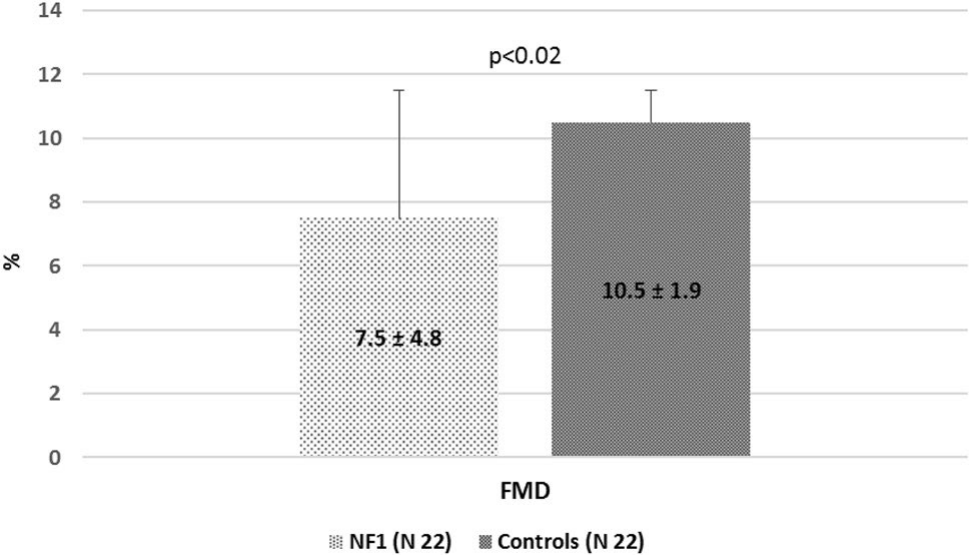
Endothelial function of brachial artery evaluated by Flow Mediated Dilation (FMD) technique and ischemic test in NF1 subjects and control subjects.

Endothelial function evaluated by the FMD technique and ischemic test was significantly lower in NF1 subjects than healthy subjects (Fig. 2), even after controlling for potential confounding factors like sex, gender, and smoke. Among NF1 subjects, 80% were Early dilators, and 20% were No dilators, while among healthy subjects, all were Early dilators. Late dilators were absent in both groups; therefore, peak FMD overlapped with FMD measured at 1 min.

**Figure 3:**
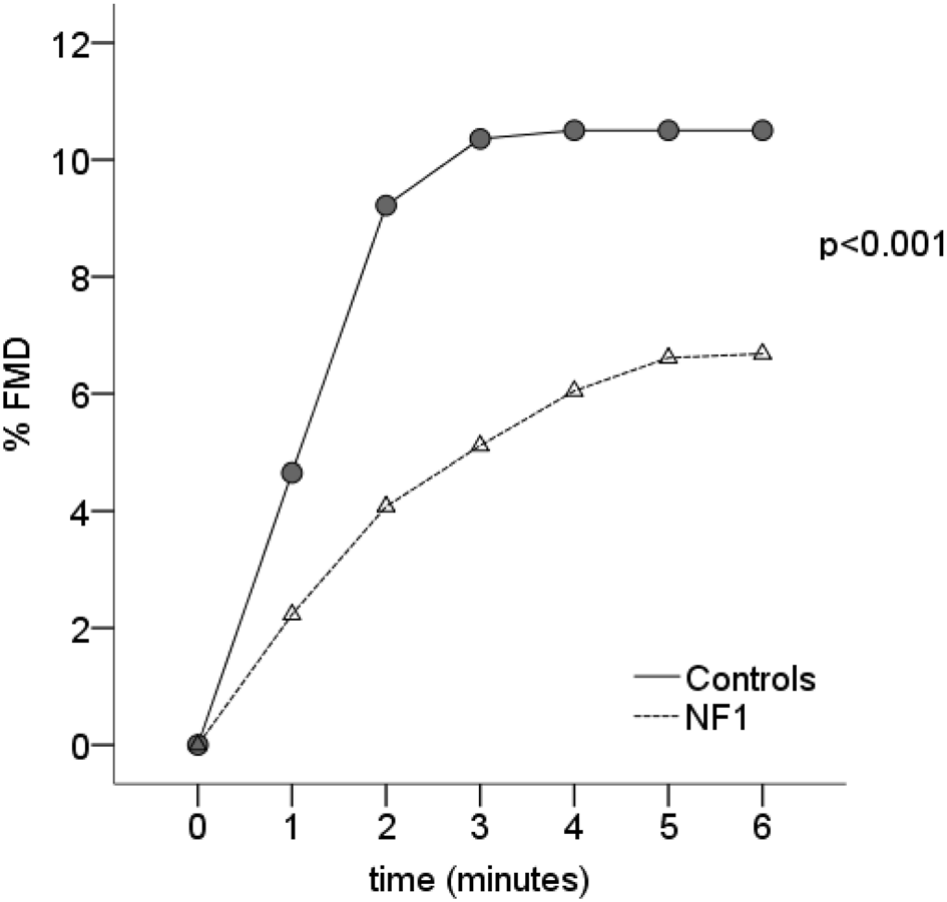
Endothelial function of brachial artery evaluated as percentage of dilation after Hand Grip Exercise in NF1 subjects and control subjects.

Arterial vasodilation evaluated by HG-EX was also significantly dumped in NF1 subjects. 17 out of 22 subjects with NF-1 agreed to perform the exercise. Maximum voluntary contraction (MVC) was similar among two groups according to sex: 36.5(15) kg in NF1 males versus 39(15) kg in controls males (*p* = 0.15); 22(2) kg in NF1 females versus 20.5(5) kg in controls females (*p* = 0.11) (data are expressed as median and IQR). Figure 3 shows the curve of brachial artery dilation expressed as a percentage of dilation during exercise. The percentages were the following respectively in Control and NF1 subjects: 1st min 4.6 ± 2.6 versus 2.2 ± 1.4%; 2nd min 9.2 ± 2.5 versus 4.1 ± 2.0%; 3rd min 10.4 ± 2.1 versus 5.1 ± 3.0%; 4th min 10.5 ± 2.1 versus 6.0 ± 3.5%; 5th min 10.5 ± 2.1 versus 6.6 ± 3.5%; 6th min 10.5 ± 2.1 versus 6.7 ± 3.4 (mixed ANOVA: main effect of groups F(1,37) = 33.01, *p* < 0.001; main effect of time, F(2,89) = 182, *p* < 0.001; interaction, F(2,89) = 14, *p* < 0.001).

In Table 2, we have displayed brachial artery diameter, and SS measured before and at the end of HG-EX. All variables significantly increased from baseline to end exercise in NF1 and Control group. No difference statistically significant was found between NF1 and Control subjects. Blood viscosity measured at a shear rate of 225/sec was also comparable between groups: NF1, 4.3 ± 0.6 versus Control subjects, 4.1 ± 0.4 cPoise (*p* = 0.23).

**Table 2:** Baseline and end-exercise brachial artery diameter, and shear stress in NF1 and control subjects.

Variable	Baseline	End exercise	*p*
**NF1 (n13)**			
Brachial artery diameter (mm)	3.1 ± 0.5	3.3 ± 0.4	0.001*
PSS (dyne/cm^2^)	65 ± 17	89 ± 29	0.02^
MSS (dyne/cm^2^)	14 ± 7	45 ± 4	0.02^
**Control subjects (n22)**			
Brachial artery diameter (mm)	3.3 ± 0.4	3.7 ± 0.4	0.001*
PSS (dyne/cm^2^)	53 ± 17	76 ± 25	0.001*
MSS (dyne/cm^2^)	7 ± 3	35 ± 14	0.001*

Data are displayed as mean ± SD.

*NF1* neurofibromatosis 1, *PSS* peak shear stress, *MSS* mean shear stress.

*p = 0.001 versus baseline; ^*p* = 0.02 versus baseline.

No significant differences regarding traditional echocardiographic parameters of LV systolic function were observed between NF1 subjects and healthy controls (Table 3). Both LV Ejection Fraction (*p* = 0.518) and Fractional Shortening (*p* = 0.270) were similar between the two study groups; however, 4.5% of NF1 patients presented LVEF below normal threshold, while no subjects from the Control group presented impaired LVEF. On the contrary, mean GLS was significantly lower in NF1 patients than controls, even if it remained within the normal range in most patients (NF1: -19.3 ± 1.7 vs. Controls: -21.5 ± 2.7, *p* = 0.008). According to reference ranges, only 18% of NF1 patients had formally impaired GLS values^18^. No subjects from the Control group presented impaired GLS values. Diastolic function was assessed in all included subjects, showing no significant differences in E/A (1.4 ± 0.45 vs. 1.3 ± 0.10; *p* = 0.365) and E/E′ (6.7 ± 2.0 vs. 6.5 ± 0.6; *p* = 0.682) ratios between NF1 patients and Control subjects.

**Table 3:** Cardiac assessment in NF1 and control subjects.

Variable	NF1 (n 22)	Controls (n 22)	*p*
LVEDD/BSA (mm/mm^2^)	25.5 ± 3.2	26.2 ± 1.5	0.407
LVESD/BSA (mm/mm^2^)	15.7 ± 2.3	15.5 ± 1.7	0.852
IVS/BSA (mm/mm^2^)	5.3 ± 1.0	4.2 ± 3.6	0.001
Ejection fraction (%)	66.3 ± 5.7	65.8 ± 4.0	0.518
Fractional shortening (%)	38.8 ± 4.7	40.6 ± 3.4	0.270
GLS (%)	− 19.3 ± 1.7	− 21.5 ± 2.7	0.008

Data are displayed as mean ± SD.

*NF1* neurofibromatosis 1, *LVEDD* left ventricle end diastolic diameter, *BSA* bosy surface area, *LVESD* left ventricle end systolic diameter, *IVS* InterVentricular Septum, *GLS* global longitudinal strain.

## Discussion

The present study results demonstrate that subjects with NF1 have functional and structural peripheral vascular abnormalities and systolic cardiac impairment in the absence of cardiovascular risk factors or cardiovascular diseases.

We have observed an impaired flow-mediated dilation in NF1 subjects evaluated with both ischemia and exercise stimuli. It is well established that among endothelium’s functions, tone and vessel diameter regulation is probably the best known. Abnormalities in this regulatory mechanism promote atherosclerosis development^21^, which has been observed in NF1 vasculopathy.

HG-EX and reactive hyperemia after ischemia have both demonstrated endothelial dysfunction. Exercise induces a sustained and more physiological shear stress profile compared with reactive hyperemia^16^: indeed it has been shown that HG-EX FMD can detect early abnormalities in endothelial function not otherwise found with reactive hyperemia in specific categories of subjects (e.g., young obese, smokers)^22,23^. However, it is necessary to emphasize that the lack of WSS data during forearm ischemia makes it challenging to distinguish between brachial artery endothelial dysfunction and reduced stimulus for downstream microvascular damage.

The present finding is at odds with the only other report on endothelial function in the literature. However, it must be emphasized that in the work of Rodrigues et al., the method used for the evaluation of endothelial function was completely different^24^.

In addition to endothelial dysfunction, we have found that subjects with NF1 have a significantly higher common carotid artery wall thickness than healthy control subjects. Although IMT was approximately 600 microns, which is far below the value considered indicative of preclinical carotid atherosclerosis, it is however, suggestive of alteration of the arterial wall. These results are in line with previous histological findings that demonstrated intima proliferation and the thickening of the media in the arterial wall of NF1 subjects^25^.

Our findings of early asymptomatic alterations in cardiac function deserve some comments as well. Both morphologic and functional cardiac alterations were previously reported in young patients with NF1 with hypertension^26,27^. However, a later study describing cardiac characterization of NF1 with or without large deletions of the NF1 gene, reported no impairment of cardiac systolic or diastolic function^28^. In this regard, our results demonstrate for the first time that NF1 patients present lower cardiac function values than controls, even in the absence of concomitant cardiovascular diseases. We used the more sophisticated echocardiographic technique so far to assess left ventricular function, the myocardial strain imaging, which is less operator-dependent and less sensitive to variations in systemic blood volume^29^. Although modest in magnitude, this finding is original and might have relevant implications in the clinical management of these subjects. Previous studies did not find any significant alteration in LV systolic function in NF1 patients. That is probably related to the use of conventional echocardiographic techniques (i.e., the EF) for the assessment of cardiac contractility, which was less sensitive and more operator dependent. In contrast, strain describes the myocardium's deformation during the cardiac cycle in the longitudinal, circumferential, and radial planes^28^. It has been previously demonstrated that GLS improves the detection of systolic dysfunction beyond LVEF. Besides, reduced GLS has a consistent independent association with adverse outcomes, and its use now is well justified for risk evaluation^30^.

Our findings demonstrate that early alterations in cardiac function can be found in young NF1 patients, even in the absence of cardiovascular symptoms or diseases as hypertension, which is one of the most common cardiac complications occurring in neurofibromatosis, or congenital heart disease, whose prevalence among NF1 patients ranges from 0.4 to 6.4. Although the pathophysiological mechanisms underlying these alterations are not clear, they can be at least partially of vascular origin. Indeed, patients with NF1 we have recruited in the study showed cardiac and peripheral artery abnormalities. Our results are in line with histologic evidence showing specific vascular remodeling patterns, such as intimal proliferation, thickening of the media, fragmentation of elastic tissue, and dysplasia of the small vessels^6^. These alterations may lead to impaired vascular compliance and cardiac myofibrillar dysplasia, as elsewhere reported^31^.

Several questions are still open regarding the molecular mechanisms underlying the pathological features of NF1 patients, such as vascular remodeling or myocardial dysfunction and remodeling. Among the most interesting observations, some are worth mentioning as multiple research groups have independently documented them. Partial or complete loss of the NF1 gene results in increased PKCδ-mediated p47phox phosphorylation through the activation of p21(Ras), resulting in increased NADPH oxidase 2 activity, alterations in the redox balance and pro-inflammatory effects^32–34^. Acceleration of Ras activity also has an impact on multiple kinases of its downstream signaling pathways, including Erk and Akt, both of which are involved in the modulation of the phenotype of cells making up the vascular wall in NF1 heterozygous (Nf1^+/−^) animal model^35,36^. Nf1^+/−^ mutant mice develop a thick neointima, resembling human arterial disease. Neurofibromin is a known downstream element of the TATA Binding Protein-related factor 2 (TRF2) axis. It is involved in both cardiac development and myocardial fibrosis, which might explain the lower GLS we measured in NF1 patients compared to controls, even though still within the normality range^37,38^. Besides, the loss of NF1-gene also affects p12(RAS) signaling in the myocardium, where it is involved in fibrosis and cardiac function^39,40^.

The present study has some strengths and some limitations: the sample size of this study is small, but the fact that we are covering a rare disease must be considered. Moreover, the study design could not consent to the blindness of ultrasound operators, configuring a potential bias. Lastly, we have not continuously measured brachial artery diameter and WSS during forearm ischemia. The maximum vasodilatation might, therefore, be underestimated. The lack of WSS during FMD does not give the certainty of the reduced FMD of NF1 patients to endothelial dysfunction of the brachial artery or a more peripheral alteration. One of the strengths is that the majority of the subjects were young without any comorbidities or cardiovascular risk factors, so our findings are due to the disease investigated. Another strength is the use of the more recent technologies in cardiology as GLS, which provides quantitative measurement and is not operator-dependent.

## Conclusion

The present study demonstrates that, in patients with NF1, alterations of vascular and cardiac function and morphology appear very early, and can be detected and followed over time. These findings might have significant clinical relevance suggesting a careful cardiovascular risk evaluation in subjects with NF1.

## References

[1] Friedman JM (2000). Insights into the pathogenesis of neurofibromatosis 1 vasculopathy. Clin. Genet..

[2] Gitler AD (2003). NF1 has an essential role in endothelial cells. Nat. Genet..

[3] Friedman JM, Friedman JM, Gutmann DH, MacCollin M, Riccardi VM (1999). Vascular and endocrine abnormalities. Neurofibromatosis: Phenotype, Natural History and Pathogenesis.

[4] Viskochil DH (1999). Neurofibromatosis: Phenotype, Natural History, and Pathogenesis.

[5] Hamilton SJ, Friedman JM (2000). Insights into the pathogenesis of neurofibromatosis 1 vasculopathy. Clin. Genet..

[6] Brannan CI (1994). Targeted disruption of the neurofibromatosis type-1 gene leads to developmental abnormalities in heart and various neural crest-derived tissues. Genes Dev..

[7] Venturin M, Bentivegna A, Moroni R, Larizza L, Riva P (2005). Evidence by expression analysis of candidate genes for congenital heart defects in the NF1 microdeletion interval. Ann. Hum. Genet..

[8] Ly KI, Blakeley JO (2019). The diagnosis and management of neurofibromatosis Type 1. Med. Clin. North Am..

[9] Stewart W, Werhessen ND, Wolf S, Werthessen NT (1979). Fluid mechanics of arterial flow. Dynamics of Arterial Flow.

[10] Gnasso A (1996). Association between intima-media thickness and wall shear stress in common carotid arteries in healthy male subjects. Circulation.

[11] Touboul PL (2007). Mannheim carotid intima-media thickness consensus (2004–2006). Cerebrovasc. Dis..

[12] Grant EG (2003). Carotid artery stenosis: Gray-scale and doppler US diagnosis—Society of radiologists in ultrasound consensus conference. Radiology.

[13] Irace C (2012). Human common carotid wall shear stress as a function of age and gender: A 12-year follow-up study. Age (Dordr).

[14] Thijssen DHJ (2019). Expert consensus and evidence-based recommendations for the assessment of flow-mediated dilation in humans. Eur. Heart J..

[15] Irace C, Padilla J, Carallo C, Scavelli F, Gnasso A (2014). Delayed vasodilation is associated with cardiovascular risk. Eur. J. Clin. Invest..

[16] Tremblay JC, Pyke KE (2018). Flow-mediated dilation stimulated by sustained increases in shear stress: A useful tool for assessing endothelial function in humans?. Am. J. Physiol. Heart Circ. Physiol..

[17] Trush A, Hoskins P, Martin K, Thrush A (2010). Spectral doppler ultrasound. Diagnostic Ultrasound, Physics and Equipment.

[18] Lang RM (2015). Recommendations for cardiac chamber quantification by echocardiography in adults: An update from the American Society of Echocardiography and the European Association of Cardiovascular Imaging. Eur. Heart J. Cardiovasc. Imaging.

[19] Sabatino J (2019). Left ventricular twist mechanics to identify left ventricular non compaction in childhood. Circ. Cardiovasc. Imaging..

[20] Sugimoto T (2017). Echocardiographic reference ranges for normal left ventricular 2D strain: Results from the EACVI NORRE study. Eur. Heart J. Cardiovasc. Imaging.

[21] Davignon J, Ganz P (2004). Role of endothelial dysfunction in atherosclerosis. Circulation.

[22] Findlay BB, Gupta P, Szijgyarto IC, Pyke KE (2013). Impaired brachial artery flow-mediated vasodilation in response to handgrip exercise induced increases in shear stress in young smokers. Vasc. Med..

[23] Slattery DJ, Stuckless TJ, King TJ, Pyke KE (2016). Impaired handgrip exercise-induced brachial artery flow-mediated dilation in young obese males. Appl. Physiol. Nutr. Metab..

[24] Rodrigues LO, Castro LL, Rezende NA, Ribeiro AL (2013). Non-invasive endothelial function assessment in patients with neurofibromatosis type 1: A cross-sectional study. BMC Cardiovasc. Disord..

[25] Greene JF, Fitzwater JE, Burgess J (1974). Arterial lesions associated with neurofibromatosis. Am. J. Clin. Pathol..

[26] Tedesco MA (2005). Early cardiac morphologic and functional changes in neurofibromatosis type 1 hypertensives: An echocardiographic and tissue Doppler study. Int. J. Cardiol..

[27] Tedesco MA (2001). Cardiac abnormalities detected by Doppler imaging in patients with neurofibromatosis type 1. Am. J. Cardiol..

[28] Nguyen R (2013). Cardiac characterization of 16 patients with large NF1 gene deletions. Clin. Genet..

[29] Potter E, Marwick TH (2018). Assessment of left ventricular function by echocardiography: The case for routinely adding global longitudinal strain to ejection fraction. J. Am. Coll. Cardiol. Imaging.

[30] Negishi K (2015). Practical guidance in echocardiographic assessment of global longitudinal strain. J. Am. Coll. Cardiol. Imaging.

[31] Norton KK, Xu J, Gutmann DH (1995). Expression of the neurofibromatosis I gene product, neurofibromin, in blood vessel endothelial cells and smooth muscle. Neurobiol. Dis..

[32] Ghoshal P (2019). Loss of GTPase activating protein neurofibromin stimulates paracrine cell communication via macropinocytosis. Redox Biol..

[33] Indolfi C (1995). Inhibition of cellular Ras prevents smooth muscle cell proliferation after vascular injury in vivo. Nat. Med..

[34] Sorrentino S (2018). Hindlimb ischemia impairs endothelial recovery and increases neointimal proliferation in the carotid artery. Sci. Rep..

[35] Bessler WK (2016). Neurofibromin is a novel regulator of Ras-induced reactive oxygen species production in mice and humans. Free Radic. Biol. Med..

[36] Stansfield BK (2014). Ras-Mek-Erk signaling regulates Nf1 heterozygous neointima formation. Am. J. Pathol..

[37] Chong JA (2005). TATA-binding protein (TBP)-like factor (TLF) is a functional regulator of transcription: Reciprocal regulation of the neurofibromatosis type 1 and c-fos genes by TLF/TRF2 and TBP. Mol. Cell Biol..

[38] Guo X (2017). Cardioprotective role of TRAF2 by suppressing apoptosis and necroptosis. Circulation.

[39] Sato F (2017). Smad3 and Bmal1 regulate p21 and S100A4 expression in myocardial stromal fibroblasts via TNF-α. Histochem. Cell Biol..

[40] Xiang FL, Guo M, Yutzey KE (2016). Overexpression of Tbx20 in adult cardiomyocytes promotes proliferation and improves cardiac function after myocardial infarction. Circulation.

